# Case Report: Successful Treatment of a Patient with Microfilaremic Dirofilariasis Using Doxycycline

**DOI:** 10.4269/ajtmh.19-0744

**Published:** 2020-02-10

**Authors:** Arno M. Lechner, Herbert Gastager, Jan Marco Kern, Birgit Wagner, Dennis Tappe

**Affiliations:** 1 Division Medizinische Mikrobiologie, Paracelsus Medizinische Privatuniversität Salzburg, Universitätsinstitut für Medizinisch-Chemische Labordiagnostik, Salzburg, Austria;; 2 General Practioner, Salzburg, Austria;; 3 Abteilung für Medizinische Parasitologie, Institut für Spezifische Prophylaxe und Tropenmedizin, Zentrum für Pathophysiologie, Infektiologie und Immunologie, Medizinische Universität Wien, Wien, Vienna, Austria;; 4 Nationales Referenzzentrum für Tropische Infektionserreger, Bernhard-Nocht-Institut für Tropenmedizin, Hamburg, Germany

## Abstract

We report the case of a 56-year-old woman with microfilaremic dirofilariasis due to *Dirofilaria repens*, which is a very rare condition in humans. Of note, just one of six large-volume blood samples of this patient was positive for microfilariae. Polymerase chain reaction (PCR) and sequencing of the parasite gene determined the geographic origin of the causative helminth. The patient was treated successfully with doxycycline. This drug was chosen because of the patient’s reluctance to the use of ivermectin and to provide an anthelmintic effect by targeting the bacterial endosymbiont *Wolbachia* present in most filarial species.

## INTRODUCTION

Dirofilariasis is an emerging mosquito-borne nematode disease caused by *Dirofilaria repens* and *Dirofilaria immitis*. The definitive hosts are dogs and cats, whereas humans may become infected aberrantly. Infective larvae are transmitted through mosquito bites and develop into adult worms in animals, which produce microfilariae that circulate in the bloodstream. In humans, infective larvae of *D. repens* usually develop into immature worms, causing subcutaneous nodules or ocular lesions. In subcutaneous dirofilariasis, nodules arise over months. Ocular dirofilariasis involves subconjunctival, intravitreal tissues or the eyelids. In both conditions, human tissue harbors preadult and adult worms. Development into adult mating worms seldom occurs in humans, and the resulting microfilaremia is even more rare. To the best of our knowledge, microfilaremia due to *Dirofilaria* spp*.* has been published only four times,^1–4^ and in these cases, definitive species identification could not be performed. In another case of infection due to *D. repens* with concomitant meningoencephalitis, microfilaremia must be proposed, as crossing the blood–brain barrier by microfilariae is the only mechanism to involve the central nervous system.^5^ Ivermectin is an antifilarial drug used in other filarial diseases such as onchocerciasis or lymphatic filariasis. However, given the rarity of microfilaremia due to *D. repens* in humans, the use of ivermectin is not well established in the literature and based on just few case reports. At least in cases, when ivermectin is contraindicated, alternative treatments are needed.

## CASE PRESENTATION

**Figure 1.**
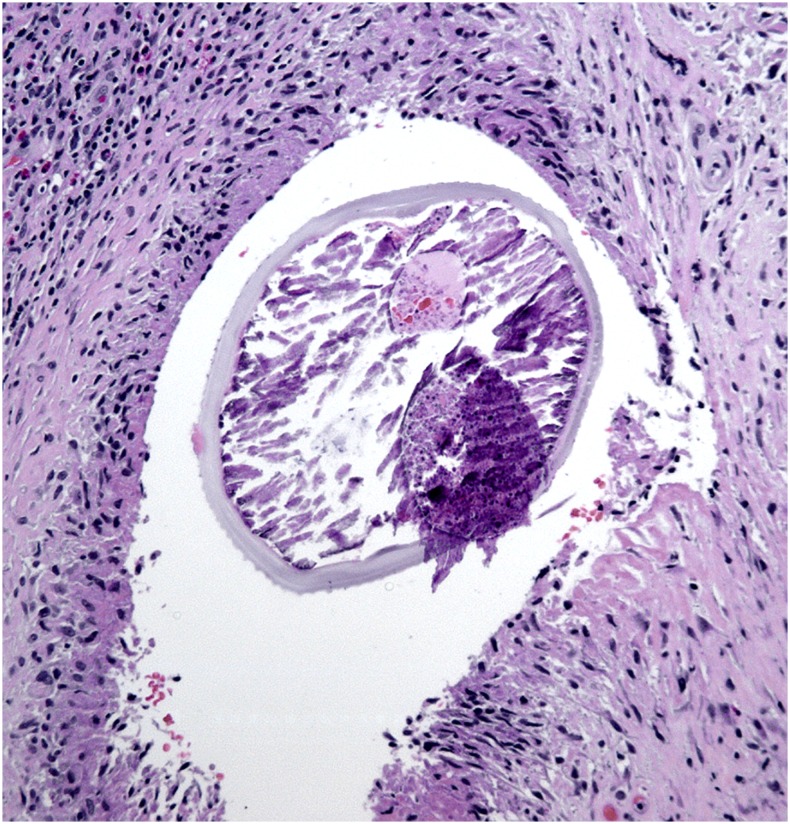
Cross section through a nematode found in an excised subcutaneous mass from the patient’s neck. The internal structures of the helminth are partly necrotic. The external ridges seen on the cuticle of the nematode are typical for *Dirofilaria repens*. This figure appears in color at www.ajtmh.org.

A 56-year-old caucasian woman living in Austria noticed indolent cutaneous swellings over a period of 9 months, the first located at the left thigh, the second at the right lower abdomen, and finally, in the right retromandibular area, each variable in terms of persistence and size (2 cm–8 cm). Two weeks after emergence of the first swelling in February 2018, the patient complained of flu-like symptoms lasting for a few days and the swelling located in the right retromandibular area turned into a persisting, painless, firm mass measuring 2.5 cm. She consulted her general practitioner, who diagnosed an eosinophilia of 27% (total leukocytes 6,170 G/L) and a total IgE of 415 U/(normal value < 130 U/mL). Beside other differential diagnoses, filariasis was suspected. The patient’s examination was otherwise unremarkable. Her travel history included stays in Sri Lanka in 2006 and India in 2015, for 1 month each. In addition, she reported vacations in Greece during the last few years for 3 weeks each. During that time, she had cared for pet dogs regularly. Serological testing for various filarial nematodes was performed with two different ELISA systems (*Achantocheilonema viteae* enzyme immunoassay, Bordier Affinity Products SA, Switzerland, and Filariasis ELISA, DRG Diagnostics, Marburg, Germany), yielding strongly positive results for antifilarial antibodies in both test systems. To further specify these positive results, two immunochromatographic assays for IgG4 antibodies against *Wuchereria bancrofti*, *Brugia malayi/Brugia timori* (Lymphatic Filariasis Rapid test PanLF Rapid^TM^, Reszon Diagnostics International, Subang Jaya, Malaysia) were performed. Both highly specific rapid tests gave negative results, and an infection due to *W. bancrofti*, *B. malayi*, and *B. timori* was considered unlikely. The cervical subcutaneous mass was excised, and microscopy showed cross sections through helminth structures suspicious for *Dirofilaria* sp*.*, most likely *D. repens* (Figure 1). In addition, the patient was examined for ocular or pulmonary manifestations of filariasis, without any pathological findings. The patient was negative for microfilaremia on three subsequent days at noon and at midnight by microscopy of Giemsa-stained peripheral thin and thick blood films. However, after filtering of the blood samples, four microfilariae were detected in one sample of 19 mL ethylenediaminetetraacetate (EDTA) blood drawn on the second day at noon. As identification of the microfilariae from the filter was challenging by microscopy, molecular testing using a nematode-specific 12S (rDNA) PCR was positive after scraping the filariae from the filter. The PCR was positive, and the sequence analysis of the 510-bp amplicon using BLAST (www://blast.ncbi.nlm.nih.gov) revealed 100% nucleotide homology with *D. repens* from Europe*.*

In addition to the surgical removal of the cervical nodule that contained the adult helminth, we considered a systemic anthelmintic therapy, given the presence of microfilariae and the risk of seeding to other organs. However, the patient was reluctant to the use of ivermectin after discussing potential adverse effects such as the Mazzotti reaction, which is characterized by chills, fever, lymphadenitis, headache, myalgia, arthralgia, tachycardia, and sometimes hypotension and shock due to disintegration of the helminths during therapy.^6,7^ We discussed the use of doxycycline instead targeting *Wolbachia*, a bacterial endosymbiont present in most filarial species.^8^ The patient was treated after informed consent with doxycycline 100 mg bid for 6 weeks. She reported minor gastrointestinal discomfort, but no serious side effect was encountered. From the beginning of anthelminthic therapy over the next 3 months, total IgE concentration dropped from 314 U/mL to 214 U/mL. Six months after completing therapy, blood eosinophils reached normal values and no new subcutaneous nodules or ocular involvement were detected subsequently.

## DISCUSSION

Dirofilariasis is an emerging mosquito-borne parasitic disease due to *D. repens* and *D. immitis*. Climate changes with global warming, changes in human behavior toward pets, and other human interventions, which influence population and distribution of dogs and cats, may contribute to the emergence of human dirofilariasis.^9^ Until 2012, 586 subcutaneous/ocular and 33 pulmonary cases of dirofilariasis have been reported from the European Union, primarily from Mediterranean countries.^10^ However, during the past 15 years, more autochthonous human cases have been reported from central Europe, one case of subcutaneous dirofilariasis from Austria,^11^ the first autochthonous case in Germany in 2014,^12^ and five more recent cases from southern Hungary.^13^ Most zoonotic filariae depend on the bacterial endosymbiont *Wolbachia* for growth, development, fertility, and survival,^14^ and therefore, it represents a potential target for therapeutic interventions. It has been shown recently that *Wolbachia* was present in 40 of 49 canine blood and adult nematode samples collected in Lithuania.^15^ Doxycycline is effective in filarial diseases such as onchocerciasis and lymphatic filariasis through eliminating *Wolbachia*, resulting in a long-term embryostatic effect and sterility of adult female worms, with a sustained reduction in microfilarial loads.^16,17^ Given the rarity of microfilaremia due to *D. repens*, ivermectin is not well established, based on just few case reports as aforementioned. Targeting *Wolbachia* in the treatment of filarial diseases has been envisaged for a wider scope of applications.^8^ This is why we decided to use doxycycline in this case, which was obviously an effective treatment.

In conclusion, this case is remarkable for the following reasons. First, microfilaremia due to *D. repens* is a very rare condition in humans. Considering the circumstance that in our patient, just one of six large-volume EDTA blood samples was positive for microfilariae, we assume that microfilaremia may be missed easily in comparable human cases. Second, by the use of molecular techniques (i.e., PCR and sequencing of a parasite gene), the geographic origin of the causative helminth could be determined in this patient, who is a frequent traveler. Third, given the scarce knowledge of anthelmintic therapy in patients with microfilaremic dirofilariasis, doxycycline may be a therapeutic off-label option, at least in cases, when the use of ivermectin is contraindicated or refused.
